# First-in-Humans Evaluation of a PD-L1–Binding Peptide PET Radiotracer in Non–Small Cell Lung Cancer Patients

**DOI:** 10.2967/jnumed.121.262045

**Published:** 2022-04

**Authors:** Xin Zhou, Jinquan Jiang, Xue Yang, Teli Liu, Jin Ding, Sridhar Nimmagadda, Martin G. Pomper, Hua Zhu, Jun Zhao, Zhi Yang, Nan Li

**Affiliations:** 1 Key Laboratory of Carcinogenesis and Translational Research (Ministry of Education/Beijing), Department of Nuclear Medicine, NMPA Key Laboratory for Research and Evaluation of Radiopharmaceuticals (National Medical Products Administration), Peking University Cancer Hospital & Institute, Beijing, China;; 2 Key Laboratory of Carcinogenesis and Translational Research (Ministry of Education/Beijing), Department I of Thoracic Oncology, Peking University Cancer Hospital & Institute, Beijing, China; and; 3 The Russell H. Morgan Department of Radiology and Radiological Science, Johns Hopkins University School of Medicine, Baltimore, Maryland

**Keywords:** PD-(L)1, PET/CT, immune checkpoint inhibitor, non–small cell lung cancer, pembrolizumab

## Abstract

^68^Ga-NOTA-WL12 is a peptide-based PET imaging agent. We conducted a first-in-human study of ^68^Ga-NOTA-WL12 for PET to study the in vivo biodistribution, metabolism, radiation dosimetry, safety, and potential for quantifying programmed death ligand-1 (PD-L1) expression levels in patients with advanced non–small cell lung cancer (NSCLC). **Methods:** In vitro assessment of the PD-L1 expression and cellular uptake of ^68^Ga-NOTA-WL12 was performed, followed by in vivo evaluation of ^68^Ga-NOTA-WL12 uptake in mouse models with tumors. Nine patients with NSCLC with lesions expressing PD-L1 were enrolled and monitored for adverse events during the study. ^68^Ga-NOTA-WL12 and paired ^18^F-FDG PET/CT imaging were performed. Uptake (SUV, SUL [SUV_lean_], and kBq/mL) values of tumors and normal organs were obtained. Radiopharmaceutical biodistribution, radiation dosimetry, and the relationship of tumor uptake to PD-L1 expression were evaluated. Follow-up ^18^F-FDG PET/CT was performed in patients who had undergone treatment with a combination of pembrolizumab with chemotherapy. **Results:** ^68^Ga-NOTA-WL12 exhibited PD-L1–specific uptake in vitro and in PD-L1–positive tumors in vivo. ^68^Ga-NOTA-WL12 PET imaging proved safe with acceptable radiation dosimetry. Physiologic tracer uptake was mainly visible in the liver, spleen, small intestine, and kidney. Tumors were clearly visible, particularly in the lungs, with a tumor-to-lung ratio of 4.45 ± 1.89 at 1 h. One hour was a suitable time point for image acquisition because no significant differences were noted in tumor-to-background ratios between 1 and 2 h. A strong, positive correlation was found between tumor uptake (SUV_peak_) and PD-L1 immunohistochemistry results (*r* = 0.9349; *P* = 0.002). ^68^Ga-NOTA-WL12 and ^18^F-FDG PET studies suggest that PD-L1 PET before therapy may indicate the therapeutic efficacy of pembrolizumab plus chemotherapy combination treatment. **Conclusion:** Our first-in-human findings demonstrate the safety and feasibility of ^68^Ga-NOTA-WL12 for noninvasive, in vivo detection of tumor PD-L1 expression levels, indicating potential benefits for clinical PD-L1 therapy.

## Visual Abstract

**Figure:**
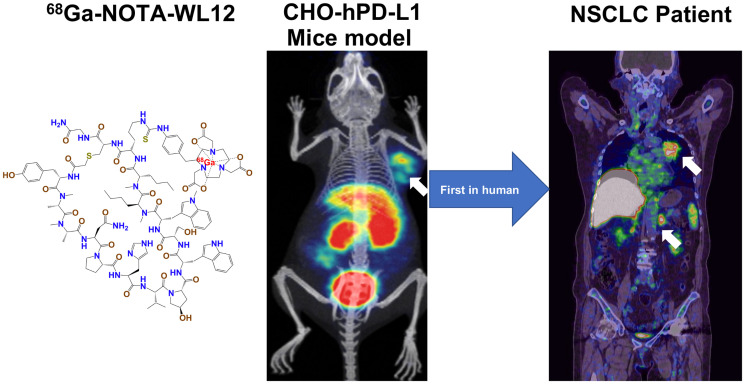

Treatment of non–small cell lung cancer (NSCLC) has advanced considerably over the past 40 y. In addition to the advent of molecularly targeted therapies, inhibition of immune checkpoints using anti–programmed cell death (ligand)-1 (PD-[L]1) monoclonal antibodies has revolutionized the management of patients with advanced NSCLC (1–6).

Therapeutics targeting the PD-(L)1 axis are now a first-line option for advanced NSCLC without genetic aberrations (7). Numerous clinical studies have shown that PD-L1 expression identified NSCLC patients who are most likely to respond to immunotherapy, such as pembrolizumab (7*,*^8^). U.S. Food and Drug Administration–approved PD-L1 assessment using immunohistochemistry and its interpretation is often based on a single biopsy or several small biopsies, which poorly represent the heterogeneity of PD-L1 expression within and between patients (9*,*^10^). Additionally, tumor biopsy is not always practical when the lesion site is inaccessible. These limitations indicate a need for tools to detect PD-L1 levels in the whole body to improve our understanding of the response of NSCLC to therapies targeting the PD-(L)1 axis.

PET enables quantitative, real-time, noninvasive assessment of target expression levels and dynamics in the whole body (11*,*^12^). Recently, several studies have investigated molecular imaging of PD-L1 expression. Biologics, such as radiolabeled antibodies and adnectin-derived small protein radiotracers, have shown promise in early phase clinical trials (13–18). High-affinity, low-molecular-weight radiotracers labeled with ^64^Cu, ^68^Ga, and ^18^F have been developed and shown to detect graded levels of PD-L1 expression in vivo in preclinical models of several cancer types, including NSCLC (13–15*,*^19^*,*^20^). One of those agents, WL12, is a high-affinity PD-L1–binding small peptide labeled with ^68^Ga. ^68^Ga-WL12 proved a suitable scaffold for imaging PD-L1 expression in preclinical studies with PET (19). The tractable pharmacokinetics and high-contrast PD-L1–specific images exhibited by ^68^Ga-WL12 within 60 min of injection indicate the potential for further evaluation and clinical translation. Here, we report the first-in-humans evaluation of a peptide-based PET imaging agent derived from WL12, ^68^Ga-NOTA-WL12 (Supplemental Fig. 1A; supplemental materials are available at http://jnm.snmjournals.org), as well as its safety, radiation dosimetry, and imaging characteristics, to compare PET imaging with immunohistochemistry and therapy evaluation in patients with advanced NSCLC.

## MATERIALS AND METHODS

### General

This was a prospective, phase I, open-label, nonrandomized, diagnostic imaging study in advanced NSCLC patients (*n* = 9) between March 2020 and September 2020 (trial registration ID NCT04304066). The Medical Ethics Committee of Peking University Cancer Hospital (2019 KT62) approved this study. Oral and written informed consent was obtained from all the participants.

NOTA-WL12 was custom synthesized and provided by Chinapeptides. Briefly, to radiolabel NOTA-WL12, ^68^GaCl_3_ (925–1,110 MBq) was mixed with 195 μL of 1 *M* (pH 8.5) sodium acetate buffer and reacted with NOTA-WL12 (30 μg) at 60°C for 15 min. The final product was purified using a Sep-Pak (Waters) and obtained in >99% radiochemical purity by high-performance liquid chromatography. Details regarding the production, quality control, and murine radiotoxicity of ^68^Ga-NOTA-WL12 can be found in the supplemental materials (Supplemental Table 1).

Patient inclusion criteria were being clinically diagnosed with NSCLC, with lesions expressing positive PD-L1; and having an Eastern Cooperative Oncology Group performance score of 0–2. Exclusion criteria were severe liver or kidney dysfunction and chemoradiotherapy or targeted therapy before PET/CT scans. PD-L1 expression in available lesions was evaluated by immunohistochemistry using antibody clone 22C3 (Dako Denmark A/S; catalog M3653). Dako PD-L1 immunohistochemistry 22C3 pharmDx is an approved screening criterion for pembrolizumab application in NSCLC (21*,*^22^). Positive PD-L1 expression was defined as a tumor proportion score (TPS) ≥ 1%, whereas high PD-L1 expression was defined as a TPS ≥ 50%.

### Flow Cytometric Analysis and Small-Animal PET

The Chinese hamster ovary (CHO) cell line was obtained from American Type Culture Collection. A CHO cell line with constitutive PD-L1 expression (CHO-hPD-L1) was generated in our laboratory and described previously (23). Details of the cell culture, flow cytometry analysis for PD-L1 expression, and in vitro assays are provided in the supplemental materials.

For PET imaging, nonobese diabetic (NOD)/severe combined immunodeficient (SCID) mice bearing CHO-hPD-L1 or CHO tumors were intravenously injected with approximately 7.4 MBq (∼0.17 μg) of ^68^Ga-NOTA-WL12 in 200 μL of saline, and PET images were acquired at 30, 60, and 120 min. To establish in vivo specificity, animals were coinjected with 50 μg of unlabeled WL12. Image analysis and PD-L1 immunohistochemistry of the CHO-hPD-L1 and CHO tumors are described in the supplemental materials.

### PET/CT

Patients were injected intravenously with ^68^Ga-NOTA-WL12 (1.9–3.7 MBq/kg). To determine the optimal peptide dose required to obtain high-contrast images, an escalated nonradiolabeled WL12 mass dose was coadministered with the imaging agent. The first 2 patients received 0 μg, the next 3 received 5 ug, then 60 ug, and the final group of 3 received 120 μg. Because patients coadministered with 120 μg of WL12 showed lower uptake in the liver, the last 4 patients were coinjected with 120 μg of WL12. The first patient underwent a dynamic scan at 6 time points in 1 h. The remaining 8 patients underwent PET/CT at 1 h after injection, and 6 of those were also scanned at 2 h after injection (2 patients were unable to tolerate the full 2-h examination). Imaging was performed using a Biograph mCT Flow 64 scanner (Siemens) (120 kV; 146 mAs; slice: 3 mm; matrix: 200 × 200; iterations:2; subsets: 11; filter: 5 mm gaussian), continuously moving the patient bed at a speed of 1.5 mm/s to cover the entire body, from the top of the skull to the middle of the femur. Images were reconstructed with ordered-subset expectation maximization. CT reconstruction used a standard method with a 512 × 512 matrix and a layer thickness of 3–5 mm. CT data were used to correct the PET images for attenuation. Vital signs, laboratory studies, and electrocardiograms were obtained before injection, during the screening period, and 2 d after PET/CT. All patients underwent paired ^18^F-FDG PET/CT scans (1 h after injection) within a week after ^68^Ga-NOTA-WL12 PET/CT using the same imaging system. Three patients repeated the ^18^F-FDG PET/CT examination within 3 wk after combination therapy including pembrolizumab and chemotherapy.

### Radiation Dosimetry

Data from 4 patients with 120 μg of coinjected WL12 were used for ^68^Ga-NOTA-WL12 dosimetry analysis, and the heart contents, lungs, liver, spleen, pancreas, kidneys, uterus, urinary bladder contents, and body remainder were selected as source organs. The volumes of the source organs manually drawn on CT images were calculated, and their mean counts/mL (kBq/mL) were determined from PET images at 1 and 2 h time points. Dosimetry was estimated using OLINDA/EXM software (version 2.0; Hermes Medical Solutions AB). Detailed procedures are provided in the supplemental materials.

### Image Analysis

Images were analyzed by 2 experienced nuclear medicine physicians. The uptake parameters of major organs, tissues, and lesions were obtained to determine the organ biodistribution. SUV_lean_ (SUL) was obtained by standardizing SUV to the body mass, which is less dependent on body habitus (24). No significant difference was observed in the coefficients of variation between SUL_mean_ and SUV_mean_ (Supplemental Fig; 2A; Supplemental Table 2). Accordingly, we used SUL_mean_ and percentage of injected activity to describe radiopharmaceutical uptake in normal organs. To compare the uptake differences between the liver, spleen, small intestine (SI), and kidney among patients coadministered with different doses of WL12, multisite measurement was applied, and the uptake was calculated as an average of the number of patients corresponding to different dose administrations separately (Supplemental Fig. 3). To analyze normal organ uptake at different time points, 6 patients with 1- and 2-h imaging were involved. The maximum, peak, and mean values of SUV and SUL in biopsied lesions were obtained and used to correlate with PD-L1 TPS. Tumor uptake of ^68^Ga-NOTA-WL12 higher than that of blood pool (BP) was considered positive. Evaluation of the therapeutic response was based on PERCIST (24) (for ^18^F-FDG PET/CT evaluations) and RECIST 1.1 (25) (for CT evaluations) standards.

### Statistics

Differences and correlations among parameters were tested using the Wilcoxon signed-rank test, Mann–Whitney *U* test, and Spearman correlation using IBM SPSS Statistics (version 24; IBM Corp.) software. *P* values < 0.05 were considered statistically significant.

## RESULTS

### Safety Assessment

^68^Ga-NOTA-WL12 was produced with >99% purity with a specific activity of 18.5–296 GBq/μmol (Supplemental Fig. 1B). The safety parameters were measured. Murine radiotoxicity indicated that 167–200 MBq of ^68^Ga-NOTA-WL12 were safe for humans (Supplemental Fig. 4). Details can be found in the Supplemental Materials.

### In Vitro Cellular Studies and Small-Animal PET Imaging

Flow cytometry showed that the mean fluorescence intensity values of CHO-hPD-L1 were higher than those of CHO, as was cellular uptake extent (Supplemental Fig. 5).

**FIGURE 1.**
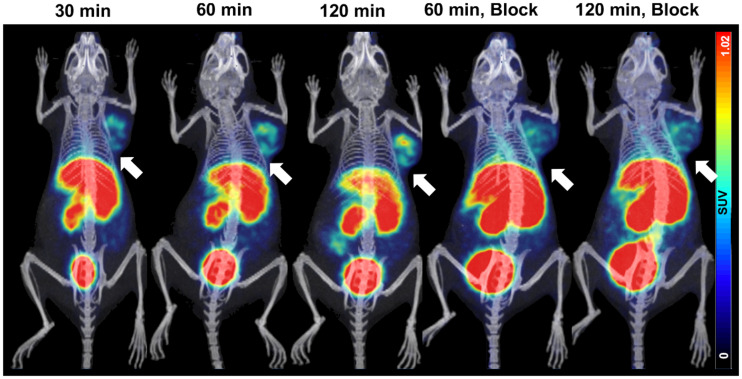
PET/CT images of NOD/SCID mice with CHO-hPD-L1 tumors at different time points after ^68^Ga-NOTA-WL12 injection and of mice receiving 50 μg amount of blocking dose.

**FIGURE 2.**
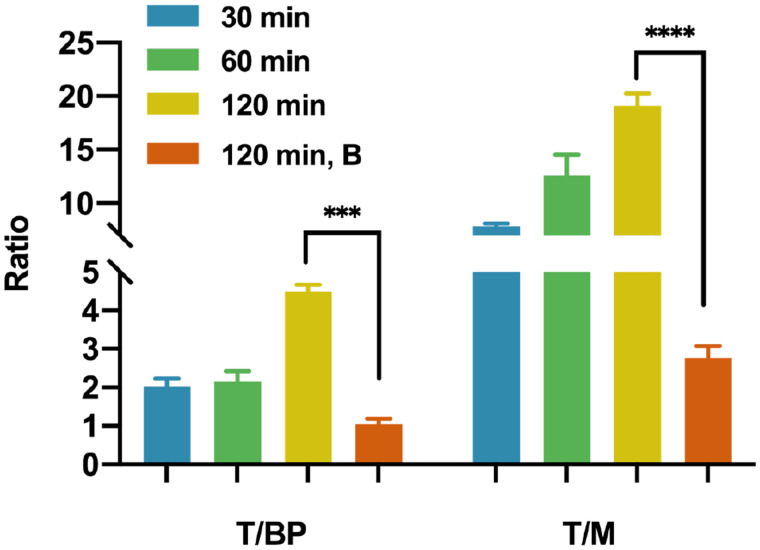
Ratios of T/BP and T/M in mice receiving ^68^Ga-NOTA-WL12 with and without blocking dose at different time points.

NOD/SCID mice bearing CHO-hPD-L1 tumors showed intense uptake of ^68^Ga-NOTA-WL12 in the tumors by 120 min (tumor–to–blood-pool uptake [T/BP]: 4.5 ± 0.2; tumor-to-muscle uptake [T/M]: 19.1 ± 1.2) (Fig. 1), whereas negative control CHO tumors showed minimal uptake (Supplemental Fig. 6A). Additionally, tumor uptake decreased with the coinjection of NOTA-WL12, indicating in vivo specificity (Fig. 2). Immunohistochemistry analysis showed high PD-L1 expression in CHO-hPD-L1 tumors (Supplemental Fig. 6B).

### Patient Characteristics

Nine patients (8 men and 1 woman; median age, 68 y; age range, 47–80 y) with histopathologically proven NSCLC (5 adenocarcinoma, 4 squamous cell carcinoma) were included. Clinical stage, therapeutic regimen, PD-L1 expression and other characteristics are summarized in Table 1. The mean administered radioactivity of ^68^Ga-NOTA-WL12 was 195 ± 30 MBq (range, 167–270 MBq).

**TABLE 1.** Patient Characteristics

Addition of Wl12 (ΜG)	Patient	Sex	Age (y)	ECOG score	Tumor type	PD-L1 expression*	Clinical stage	Tumor size (cm)	Therapy regimen
0	1	M	47	1	LUSC	8%	cT4N2M1a IVa	7.2 × 6.1	Nab-paclitaxel + carboplatin + pembrolizumab
0	2	M	72	0	LUSC	35%	cT2N3M0 IIIb	4 × 3.4	Paclitaxel + cisplatin
5	3	M	68	0	LUSC	8%	cT4N1M1a IVa	5.8 × 4.7	Nab-paclitaxel + carboplatin + pembrolizumab
60	4	M	68	1	LUAC	25%	cT4N1M0 IIIa	5.1 × 4.0	Nab-paclitaxel + carboplatin
120	5	M	80	2	LUAC	40%	cT4N3M1c IVb	5.7 × 3.9	toripalimab
	6	M	58	0	LUAC	30%	cT2aN2M1c IVb	3.1 × 2.3	Pemetrexed + carboplatin + pembrolizumab
	7	M	63	1	LUSC	25%	cT2bN2M1b IVa	4.2 × 4.1	pembrolizumab
	8	M	53	1	LUAC	35%	cT2bN2M1b IVa	3.1 × 1.9	Pemetrexed + carboplatin + sintilimab
	9	F	80	2	LUAC	80%	cT3N3M1c IVb	5.9 × 5.3	Pembrolizumab

ECOG = Eastern Cooperative Oncology Group; LUSC = lung squamous cell carcinoma; LUAC = lung adenocarcinoma.

### Safety

Nine ^68^Ga-NOTA-WL12 PET/CT examinations were performed, with no adverse or clinically detectable pharmacologic effects. No significant changes were observed in vital signs, results of laboratory studies, or electrocardiograms.

### Biodistribution

**FIGURE 3.**
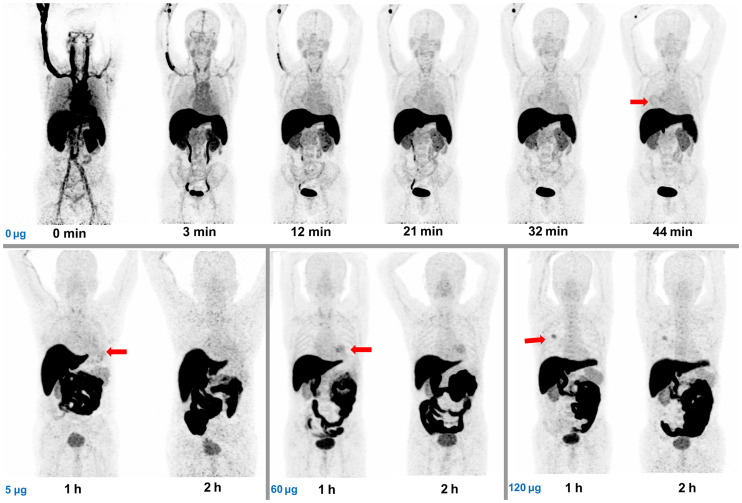
Maximum-intensity-projection imaging for biodistribution and organ uptake of ^68^Ga-NOTA-WL12 at different time points after injection administered without and with increasing precursor doses (5 μg, 60 μg, 120 μg). Primary lesions are indicated by red arrows.

Uptake of ^68^Ga-NOTA-WL12 was mainly observed in the SI and liver, followed by moderate-to-low uptake in the kidneys, tumor, and spleen, and low uptake in the lungs and bone marrow (Fig. 3). This uptake pattern was similar to that observed in preclinical studies (19*,*^20^*,*^26^*,*^27^). With an increasing precursor dose coadministered with ^68^Ga-NOTA-WL12, we observed decreased and increased uptake in the liver and SI, respectively, as well as a gradual decrease in the spleen uptake. Radioactivity uptake in the kidneys and BP at 1 h remained similar irrespective of the mass of WL12 used (Fig. 3; Supplemental Figs. 7A and 7B). Uptake observed in the liver, SI, and kidneys indicates that clearance of ^68^Ga-NOTA-WL12 was primarily through the hepatobiliary system and secondarily through renal excretion. Except for the significant increase in uptake in the SI and the significant decrease in uptake in the liver from 1 to 2 h, other normal organs showed a downward trend with no significant differences (Supplemental Figs. 7C and 7D).

### Radiation Dosimetry

Table 2 summarizes the individual organ doses and effective doses of patients administered ^68^Ga-NOTA-WL12 with120 μg of WL12 (*n* = 4) (administered dose: 224 ± 37 [range, 192–270 MBq]). The SI (3.47E–01 mGy/MBq) received the highest dose, indicating that WL12 is metabolized by hepatobiliary clearance, a characteristic observed with lipophilic agents. The intestinal absorbed dose remained well below the threshold for the human intestinal acute dose (6 Gy) (28). Radiation dosimetry was acceptable at 1.85E–02 ± 4.07E–03 mSv/MBq (4.1 mSv per patient), which is lower than the radiation dose of conventional ^18^F-FDG PET/CT (7.0–14 mSv) (29).

**TABLE 2.** Organ Observed Doses and Whole-Body Effective Doses

Organ/tissue	Absorbed dose (mGy/MBq)
Adrenals	2.60e–02
Brain	8.80e–04
Esophagus	7.43e–03
Eyes	8.88e–04
Gallbladder wall	3.21e–02
Left colon	1.52e–02
SI	3.47e–01
Stomach wall	8.41e–03
Right colon	1.26e–02
Rectum	6.83e–03
Heart wall	1.45e–02
Kidneys	3.42e–02
Liver	1.92e–01
Lungs	1.55e–02
Pancreas	2.13e–02
Prostate	4.39e–03
Salivary glands	1.09e–03
Red marrow	6.10e–03
Osteogenic cells	4.05e–03
Spleen	2.89e–02
Testes	1.16e–03
Thymus	3.85e–03
Thyroid	2.15e–03
Urinary bladder wall	1.16e–02
Total body	1.04e–02
Effective dose (mSv/MBq)	1.85e–02

### Tumor Uptake and Correlation with Immunohistochemistry

Rapid clearance of radioactivity was observed from the BP, resulting in high T/BP (and muscle) ratios. Thus, the T/BP and T/M ratios were 1.48 ± 0.42 and 1.56 ± 0.49 and 5.31 ± 1.99 and 4.87 ± 1.28 at 1 and 2 h, respectively. High contrast was also observed in the lungs with tumor-to-lung ratios of 4.45 ± 1.89 and 5.18 ± 2.27 at 1 and 2 h, respectively. No significant differences were noted in the tumor-to-background ratios (BP, lungs, and M) at 1 and 2 h (Supplemental Fig. 2B), indicating that 1 h after radiotracer administration is a suitable time point for image acquisition.

**FIGURE 4.**
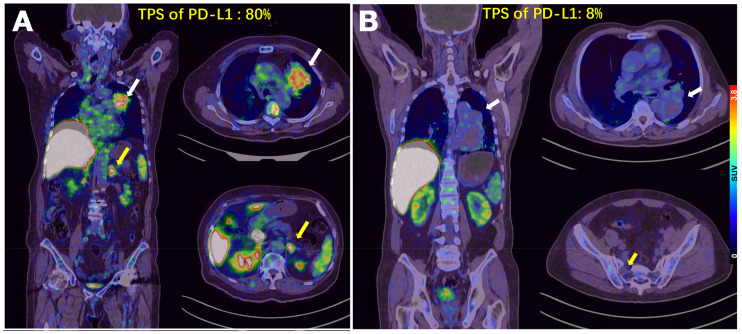
(A) Patient 9, an 80-y-old woman with advanced NSCLC and a PD-L1 TPS of 80%. SUV_max_ of primary tumor was 4.87 (white arrow) and that of left adrenal metastasis was 5.47 (yellow arrow) on ^68^Ga-NOTA-WL12 PET. (B) Patient 3, a 68-y-old man with a PD-L1 TPS of 8%. SUV_max_ of primary tumor in left lung (white arrow) and right sacral metastasis (yellow arrow) were 1.84 and 0.8, respectively, on ^68^Ga-NOTA-WL12 PET.

**FIGURE 5.**
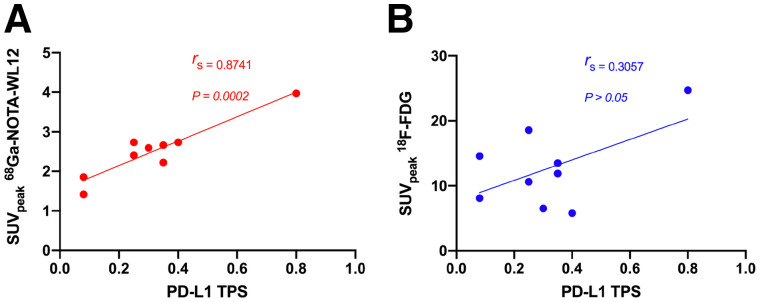
Relationship between PD-L1 expression detected by immunohistochemistry and tumor uptake (SUV_peak_) of ^68^Ga-NOTA-WL12 (*r*_s_ = 0.8741, *P* < 0.0005) (A) and ^18^F-FDG (*r*_s_ = 0.3057, *P* > 0.05) (B).

In patients with high PD-L1 expression, tumor uptake of ^68^Ga-NOTA-WL12 (TPS: 80%; SUV_max_: 4.87) was higher than that in patients with low PD-L1 expression (TPS: 8%; SUV_max_: 1.84) (Fig. 4). All calculated PET parameters of ^68^Ga-NOTA-WL12, except for the ratios of tumor uptake to BP, correlated with the corresponding PD-L1 TPS on immunohistochemistry (Supplemental Table 3) (SUV_peak_ [*r* = 0.9349, *r_s_* = 0.8741; *P* = 0.002] [Fig. 5A]). In contrast to ^68^Ga-NOTA-WL12, uptake of ^18^F-FDG in the lesions did not correlate with PD-L1 expression, with a tenuous relationship to SUV_peak_ (*r* = 0.5529, *r_s_* = 0.3057; *P* = 0.1226) (Fig. 5B).

**FIGURE 6.**
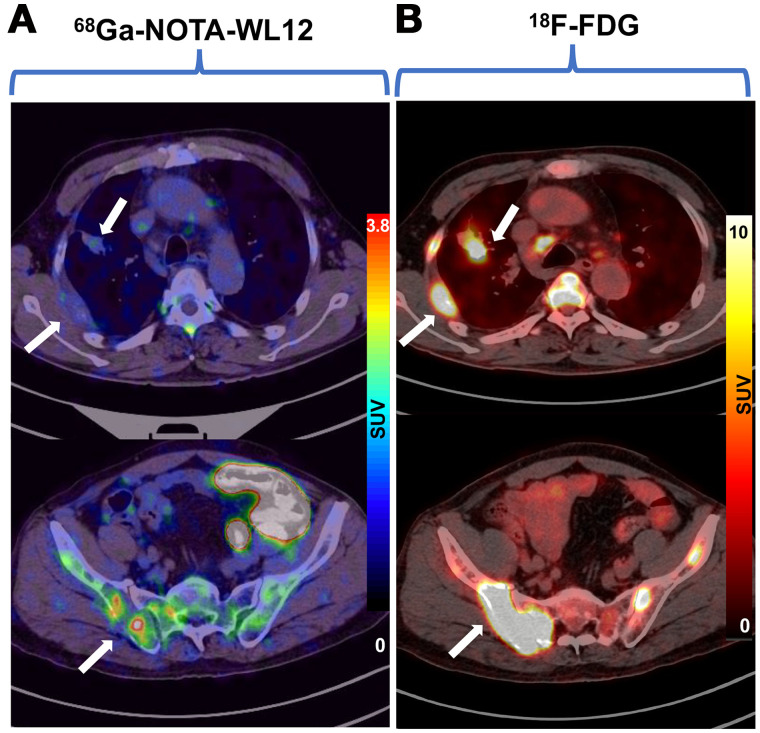
For patient 8, a 68-y-old man with lung adenocarcinoma, images showed inhomogeneous intra- and intertumoral uptake on ^68^Ga-NOTA-WL12 PET/CT (A) and high homogeneous uptake in tumors (white arrow) on ^18^F-FDG PET/CT (B).

We also noted the intra- and intertumoral heterogeneity of ^68^Ga-NOTA-WL12 uptake in some patients, reflecting the heterogeneity of PD-L1 expression reported with other PD-L1 imaging agents (Fig. 6) (13*,*^16^). The uptake of ^18^F-FDG was intense in the tumors regardless of PD-L1 expression levels, with no significant heterogeneity among or within lesions.

### Relationship of ^68^Ga-NOTA-WL12 Uptake to Therapy

**FIGURE 7.**
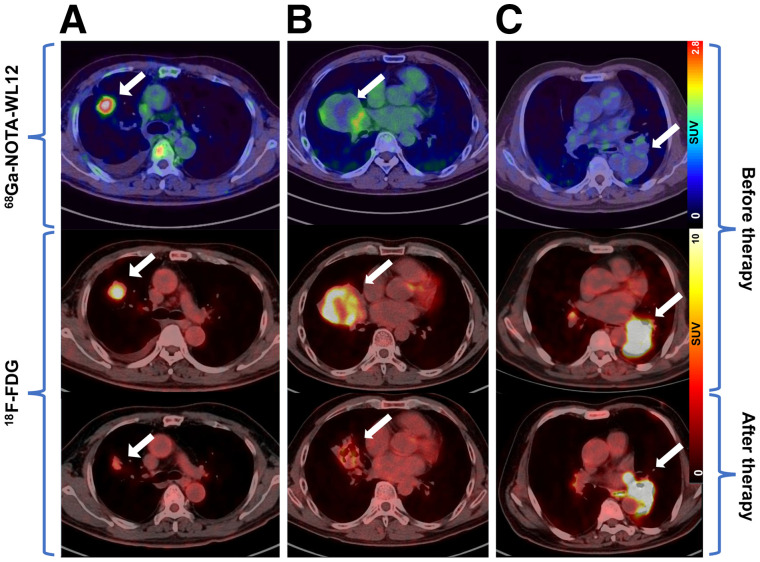
(A) Patient 6, a 58-y-old man with adenocarcinoma and a PD-L1 TPS of 30%. SUV_max_ of ^68^Ga-NOTA-WL12 was 3.05 and that of ^18^F-FDG decreased from 8.03 to 3.10. (B) Patient 1, a 47-y-old man with squamous cell carcinoma and a TPS of 8%. SUV_max_ of ^68^Ga-NOTA-WL12 was 2.21 and that of ^18^F-FDG decreased from 9.13 to 3.54. (C) Patient 3, a 68-y-old man with squamous cell carcinoma and a TPS of 8%. SUV_max_ of ^68^Ga-NOTA-WL12 was1.84 and that of ^18^F-FDG increased from 16.55 to 21.38. All lesions were indicated by white arrows.

Three patients underwent ^18^F-FDG PET/CT after combination (pembrolizumab plus chemotherapy) treatment. In patients 1 and 6, with PD-L1 TPS values of 8% and 30%, respectively, ^68^Ga-NOTA-WL12 uptake in tumors before treatment showed an SUV_max_ of 2.21 and 3.05 (Figs. 7A and 7B; Supplemental Table 4), respectively. These 2 patients were rated as partial metabolic responses (PMR, PERCIST (24)) and stable disease (RECIST 1.1 (25)).

Patient 3, with a PD-L1 TPS value of 8%, showed negative ^68^Ga-NOTA-WL12 uptake before therapy (SUV_max_ of tumor: 1.84; SUV_max_ of BP: 1.78). This patient showed increased uptake of ^18^F-FDG in the primary tumor and a new brain metastasis on the posttherapy scan, and he was further rated as progressive disease (PD, PERCIST and RECIST) (Fig. 7C; Supplemental Table 4). Thus, patients with PMR/stable disease exhibited positive uptake of ^68^Ga-NOTA-WL12 before therapy.

## DISCUSSION

We describe the first-in-humans evaluation of ^68^Ga-NOTA-WL12, a peptide-based PD-L1 imaging agent, in patients with NSCLC. We demonstrated that ^68^Ga-NOTA-WL12 is safe and effective for PET imaging of PD-L1 expression.

Inhibition of the PD-(L)1 axis has proved a remarkable success in treating patients with NSCLC (1–4). PD-L1 expression, determined by needle biopsy, is currently the only validated biomarker used as a companion diagnostic test for NSCLC patient selection for pembrolizumab therapy (22). Several studies have demonstrated variations in PD-L1 expression within patients and within tumors, due to heterogeneity of target expression (30). PD-L1 heterogeneity may still confound a single positive biopsy result, leading to inappropriate administration of therapy, contributing to the moderate correlation between PD-L1 status and survival rates (30). However, nuances in PD-L1 heterogeneity and its relevance to response are emerging. Patients with PD-L1 TPS values ≥ 50% can now be treated with pembrolizumab as a first-line option, indicating that increased PD-L1 expression is related to improved clinical outcomes (31). Patients with PD-L1 expression > 75% and > 90% have benefitted more than those with 50%–75%, suggesting that analysis and correlation of outcome data using PD-L1 expression on a continuous 0%–100% scale rather than using predefined cutoffs (≥50%) would be more accurate. These studies indicate that PD-L1 heterogeneity is an underappreciated aspect in assessing and guiding immune checkpoint therapies. Furthermore, these issues are compounded in advanced-stage NSCLC patients because a biopsy of every lesion is not feasible. Noninvasive quantification of PD-L1 levels could provide complementary information to address those challenges, as shown herein and by other groups (13*,*^16^).

Many radiolabeled probes targeting PD-L1 have been validated in preclinical models, such as antibodies, antibody fragments, small proteins, and peptides (13–17). Correspondingly, the preclinical experiments in this study confirmed that the uptake of ^68^Ga-NOTA-WL12 was highly correlated to PD-L1 expression. The accumulation of ^89^Zr-atezolizumab, a PD-L1 antibody, in patients with breast, lung, and bladder cancers showed a higher predictive value than immunohistochemistry or genomic sequencing for therapeutic response (16). Additionally, the accumulation of ^89^Zr-atezolizumab and another PD-L1 PET imaging agent, ^18^F-BMS-986192, was found to be heterogeneous between and within patients (13*,*^16^). Several studies revealed that the addition of nonradiolabeled peptide precursor reduced liver uptake through competitive metabolism; however, the excretion of ^68^Ga-NOTA-WL12 to the SI was elevated with the increase in the peptide dose. That finding differed from other saturation antibody studies (32), suggesting that the mass of ^68^Ga-NOTA-WL12 in circulation was relatively stable, and the addition of peptide would not likely affect tumor uptake. Therefore, patients with different mass doses of peptide precursor were enrolled. The correlation between uptake and therapeutic outcome, together with the inter- and intratumor heterogeneity observed with ^68^Ga-NOTA-WL12 uptake, indicated the potential to predict the effectiveness of immune therapy.

Radiolabeled antibodies such as ^89^Zr-atezolizumab and ^89^Zr-nivolumab, because of their long physical half-lives and circulation times, may encounter difficulties in delineating the changes in PD-L1 expression (13*,*^16^). Additionally, PET measures of radiolabeled antibodies do not accurately measure target expression; they are a combination of tracer exposure and target expression (16). Small-molecule radiotracers such as ^68^Ga-NOTA-WL12 provide a direct measure of the PD-L1 status within hours of radiotracer administration, likely because of better tissue penetration. Determining the characteristics of WL12 peptide binding to PD-L1 also presents opportunities to quantify the pharmacologic activity of PD-L1 antibodies within the tumor bed in a manner agnostic to the antibody type, as recently shown in preclinical models (20).

Therapeutic evaluation based on ^18^F-FDG PET/CT for lung cancer is widely recognized by physicians (24*,*^33^). Three patients in this study who received immunotherapy underwent ^18^F-FDG PET/CT for therapeutic evaluation. Patients with positive uptake of ^68^Ga-NOTA-WL12 were rated as having a PMR, and patients with a negative uptake of ^68^Ga-NOTA-WL12 were rated as having PD. Although 2 of the 3 patients shared the same PD-L1 expression level (TPS: 8%), the outcomes (PMR and PD) were quite different. These results suggest that higher ^68^Ga-NOTA-WL12 uptake in lesions may indicate a better prognosis than lower ^68^Ga-NOTA-WL12 uptake, regardless of the level of PD-L1 expression determined by immunohistochemistry. Those observations in a small number of patients merit further validation in larger patient cohorts.

A potential limitation of our study is the small number of patients involved. Nonetheless, our study is similar to other first-in-humans phase 1 studies of radiopharmaceuticals and has sufficient power to assess safety, suitable imaging time points, radiation dosimetry, and preliminary correlation of ^68^Ga-NOTA-WL12 uptake with immunohistochemistry. Another limitation of the study is that immunohistochemistry was performed only on index lesions, limiting our ability to quantify intralesional variation within a given patient.

## CONCLUSION

This first-in-humans study of ^68^Ga-NOTA-WL12, a low-molecular-weight peptide-derived imaging agent, demonstrates the feasibility and potential of quantifying PD-L1 levels in NSCLC with PET within a clinically viable time frame. ^68^Ga-NOTA-WL12 proved safe, with favorable biodistribution and radiation dose estimates similar to those of other radiopharmaceuticals. ^68^Ga-NOTA-WL12 uptake measures correlated with PD-L1 levels detected by immunohistochemistry, suggesting its suitability as a complementary diagnostic to immunohistochemistry to quantify PD-L1 levels for patient selection and therapeutic monitoring in anti-PD-L1 therapy.

## DISCLOSURE

Financial support for this study was provided by the National Science and Technology Major Project (no. 2020ZX09201023), National Natural Science Foundation of China (81671733, 81871386, and 81871387), and Beijing Excellent Talents Funding (2017000021223ZK33). Sridhar Nimmagadda and Martin G. Pomper are supported by NIH 1R01CA236616 and NIH P41EB024495; they are coinventors on a pending U.S. patent covering WL12 and, as such, are entitled to a portion of any licensing fees and royalties generated by this technology. This arrangement has been reviewed and approved by Johns Hopkins University in accordance with its conflict-of-interest policies. Sridhar Nimmagadda and Martin G. Pomper are consultants for Precision Molecular Inc., which has licensed the patent covering WL12 from Johns Hopkins University, and own equity in D&D Pharmatech, the parent company of Precision Molecular, Inc. No other potential conflict of interest relevant to this article was reported.

## Notes

KEY POINTS **QUESTION:** Can the peptide-based radiotracer ^68^Ga-NOTA-WL12 detect and represent the expression levels of PD-L1 in patients with NSCLC? **PERTINENT FINDINGS:** We demonstrated the feasibility and potential of ^68^Ga-NOTA-WL12 to quantify PD-L1 levels in preclinical models and in patients with NSCLC, with a strong relationship between tumor uptake and PD-L1 immunohistochemistry. **IMPLICATIONS FOR PATIENT CARE:** ^68^Ga-NOTA-WL12 can be used to image PD-L1 in vivo, indicating its potential as a complementary diagnostic to immunohistochemistry to quantify PD-L1 levels for patient selection and therapeutic monitoring in anti-PD-L1 therapy.
